# Supplementary material for: Effect of High-Pressure Torsion on the Microstructure and Magnetic Properties of Nanocrystalline CoCrFeNiGax (x = 0.5, 1.0) High Entropy Alloys
Source: Materials (Basel). 2022 Oct 16;15(20):7214. doi: 10.3390/ma15207214 (PMC9609683; doi:10.3390/ma15207214)
Supplement: Supplementary file 1 [file materials-15-07214-s001.zip › materials-1947405-supplementary.pdf]

Supplemental material to “Effect of High-Pressure Torsion on the Microstructure and Magnetic Properties of Nanocrystalline CoCrFeNiGa$_x$ (x = 0.5, 1.0) High Entropy Alloys”

Natalia Shkodich [1,*], Franziska Staab [2], Marina Spasova [1], Kirill V. Kuskov [3], Karsten Durst [2] and Michael Farle [2]

[1] Faculty of Physics and Center of Nanointegration (CENIDE), University of Duisburg-Essen, 47057 Duisburg, Germany
[2] Depertment of Physical Metallurgy, Technical University of Darmstadt, Alarich-Weiss-Str. 2, 64287 Darmstadt, Germany
[3] Center of Functional Nano-Ceramics, National University of Science and Technology MISIS, Moscow 119049, Russia
* Correspondence: natalia.shkodich@uni-due.de; Tel.: +49-2033-794284

S1. SPS with subsequent HPT of CoCrFeNiGa$_x$ (x = 0.5, 1.0) elemental powder blends

Figure S1 shows XRD spectra of the CoCrFeNiGa starting powder blend, SPS-consolidated and HPT-deformed bulk samples obtained from elemental CoCrFeNiGa$_x$ (x = 0.5, 1.0) powder mixtures. The Co, Cr, Fe, Ni, Ga powder mixture (Figure S1a, black) exhibits the expected strong and narrow Bragg peaks of the respective crystal structures, except diffraction-silent Ga.

The SPS of Co, Cr, Fe, Ni, and Ga$_x$ (x = 0.5, 1.0) powder blends at 1073K (Figures S1a–c, red) results in a broadening of peaks and in their overlapping due to material melting at grain boundaries (incomplete dissolution of metals). Signals at 65.68° and 100.4° correspond to the *fcc* phase. A negligible volume of a Fe-based solid solution with a *bcc* structure was also observed for SPS-consolidated samples. The diffraction peaks from initial elements are still present in the XRD pattern.

The HPT treatment of SPS-consolidated CoCrFeNiGa$_x$ (x = 0.5, 1.0) alloys led to the reduction of the crystallite size and a substantial increase in micro strains (lattice defects and dislocations) as for individual elements, as for solid solutions. Any other additional phases were not observed after HPT for both alloy compositions.

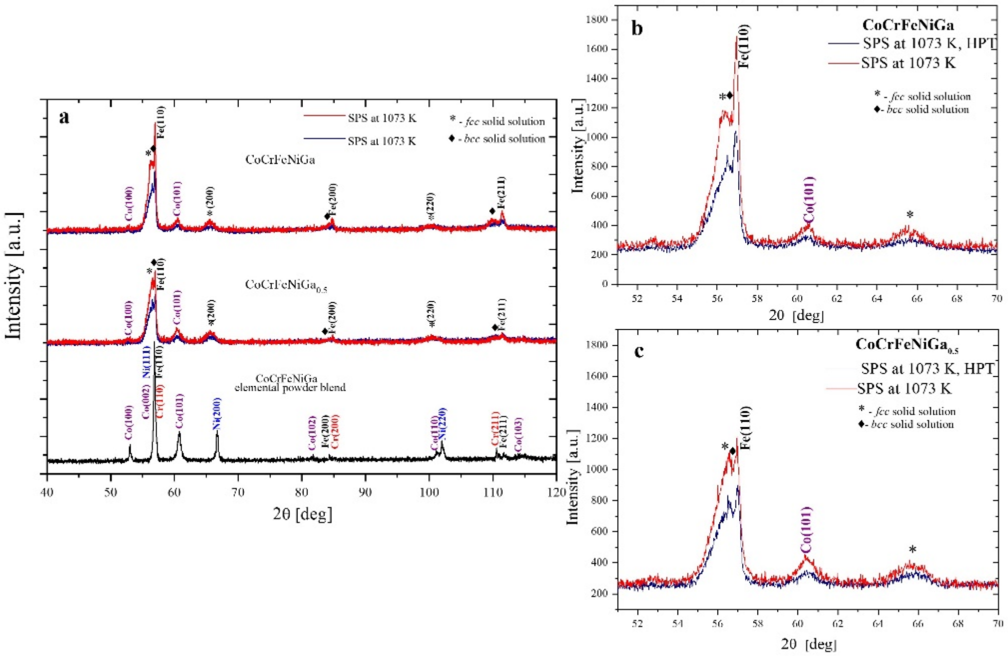

Figure S1 XRD patterns of (a) Co, Cr, Fe, Ni, and Ga elemental powder blend (black), SPS-consolidated CoCrFeNiGa$_x$ (x = 0.5, 1.0) (red) and HPT-deformed (blue) alloys; (b) SPS-consolidated CoCrFeNiGa (red) and

HPT-deformed (blue) alloy at 2θ = 51–70°; (c) SPS-consolidated $CoCrFeNiGa_{0.5}$ (red) and HPT-deformed (blue) alloy at 2θ = 51–70°

SEM images of SPS-consolidated $CoCrFeNiGa_{0.5}$ and CoCrFeNiGa alloys are presented on Fig.2a and c, respectively.

Back scatter images (Figure S2) show the distribution of elements, the Ga-rich areas (Z = 31) look brighter than the other elements: Cr (Z = 24), Co (Z = 27), Fe (Z = 26), Ni (Z = 28). The pre-alloying of elements is observed due to partial material melting at grain boundaries for SPS-consolidated samples. The distribution of principal elements for equiatomic CoCrFeNiGa and $CoCrFeNiGa_{0.5}$ alloy is shown in color-scale contour plots determined by EDX on Figure S3a and Figure S4a respectively.

Figures S2b and S2d reveal changes in the microstructure of SPS-consolidated $CoCrFeNiGa_{0.5}$ and CoCrFeNiGa alloys caused by HPT, respectively. The laminar structures composed of individual elements and their solid solutions predominantly formed at grain boundaries were observed for both materials. The SEM/EDX-mapping for the SPS-consolidated CoCrFeNiGa $CoCrFeNiGa_{0.5}$ alloys with subsequent HPT treatment are presented in Figure S3b and Figure S4b respectively.

Based on our XRD and SEM/EDX results we conclude that the SPS consolidation at 1073 K of Co, Cr,Fe ,Ni, and Ga_x (x = 0.5, 1.0) powder blends and the subsequent HPT treatment does not yield a homogeneous dissolution and the formation of HEA.

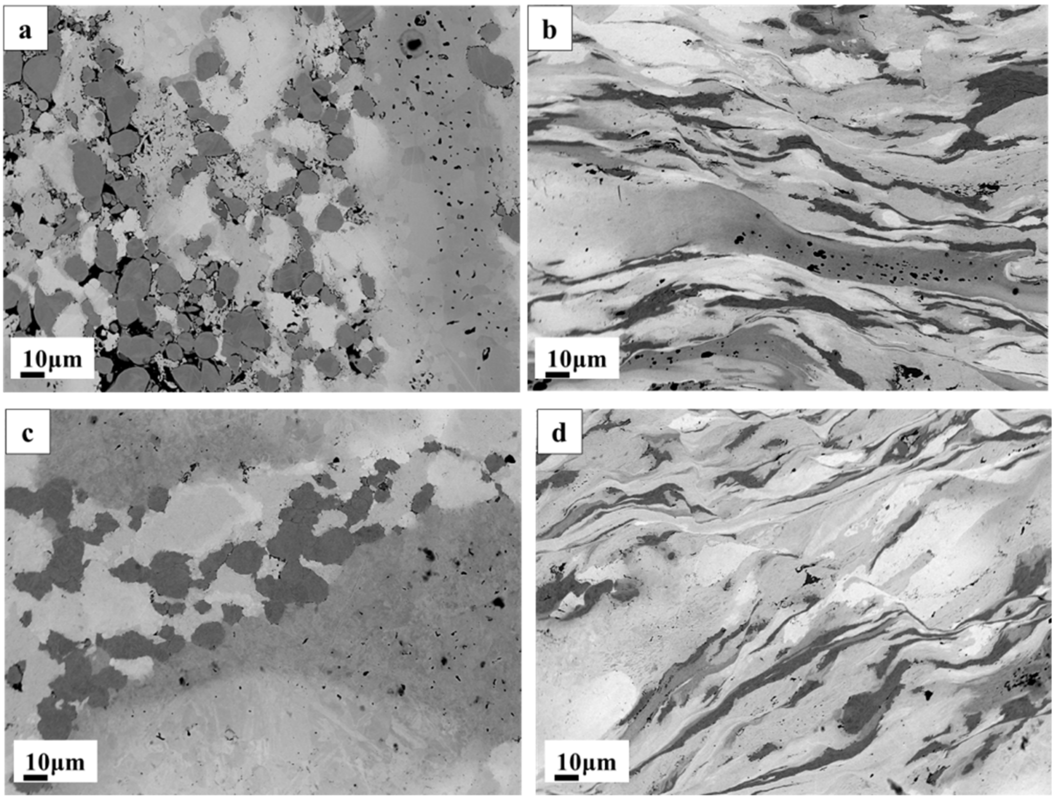

Figure S2 SEM-BSE images of SPS-consolidated samples: (a) and (b) CoCrFeNiGa alloy before and after HPT respectively; and (c) and (d) $CoCrFeNiGa_{0.5}$ before and after HPT, respectively.

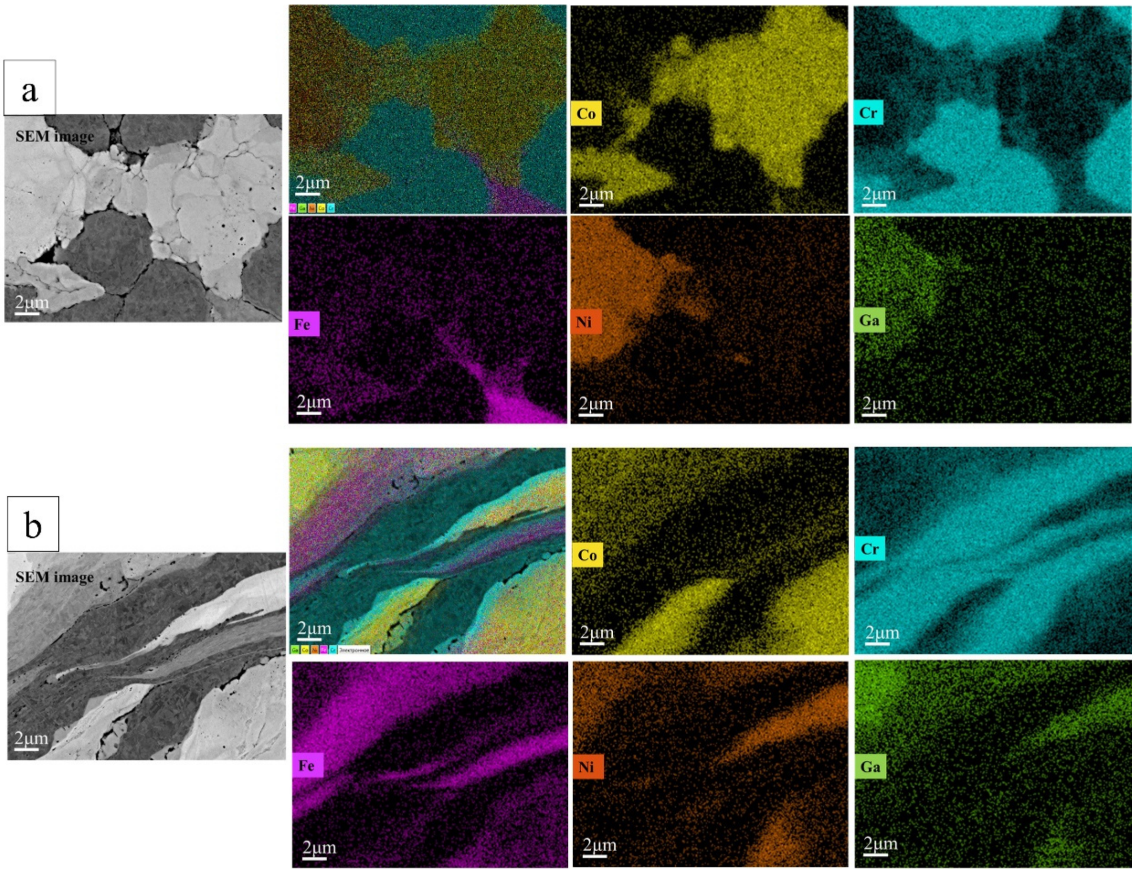

Figure S3. SEM images and EDX-mapping of the cross-section of bulk CoCrFeNiGa alloy sintered from non-milled powder blend by SPS at 1073 K (a) with subsequent HPT treatment (b).

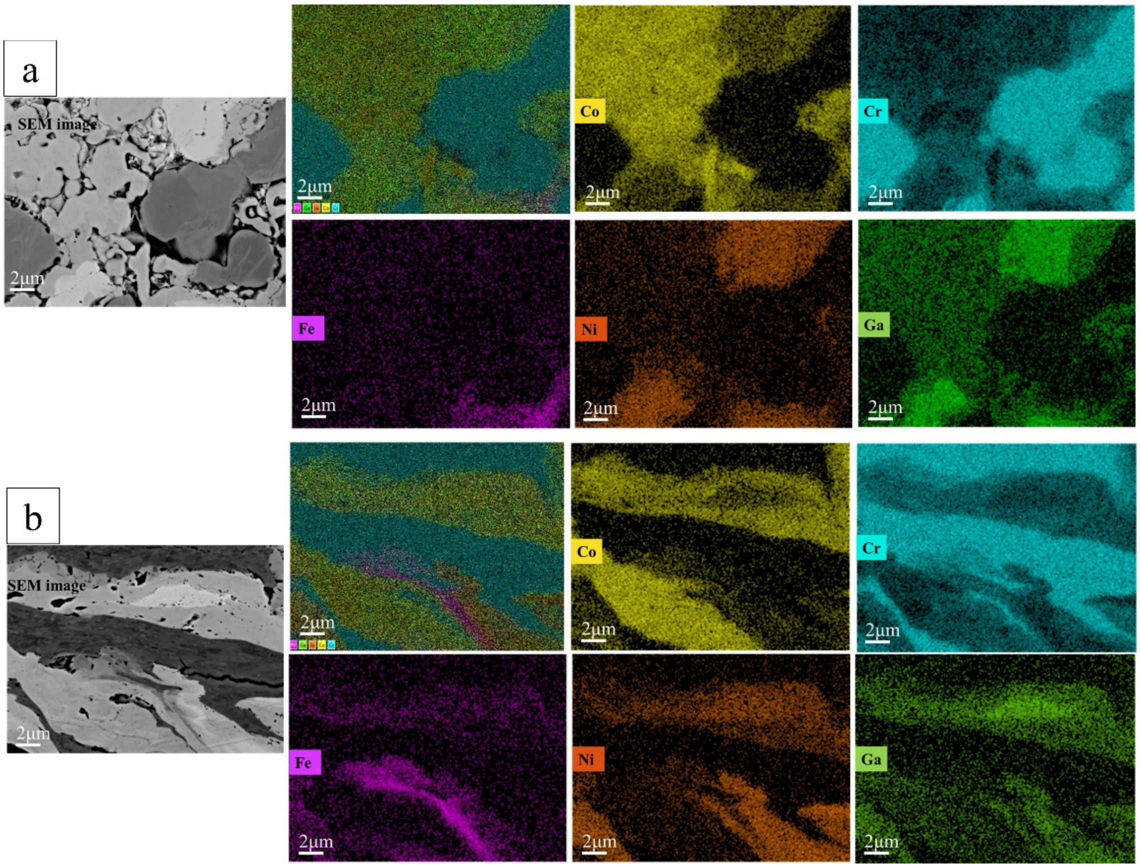

Figure S4. SEM images and EDX-mapping of the cross-section of bulk $CoCrFeNiGa_{0.5}$ alloy sintered from non-milled powder blend by SPS at 1073 K (a) with subsequent HPT treatment (b).

S2. Mechanical properties (Vickers microhardness) of SPS-consolidated and HPT-deformed CoCrFeNiGax (x = 0.5, 1.0) alloys from elemental powder blends

The dependence of the Vickers hardness (H_v) before and after HPT for SPS-consolidated $CoCrFeNiGa_x$ (x = 0.5, 1.0) samples obtained from elemental powder blends are presented in Fig. 5. SPS-consolidated samples produced from the elemental powders have the lowest H_v for both compositions (Figure S5, black). This is expected, since the SPS of constituent elements at 1073K cannot lead to a complete dissolution and a formation of HEA.

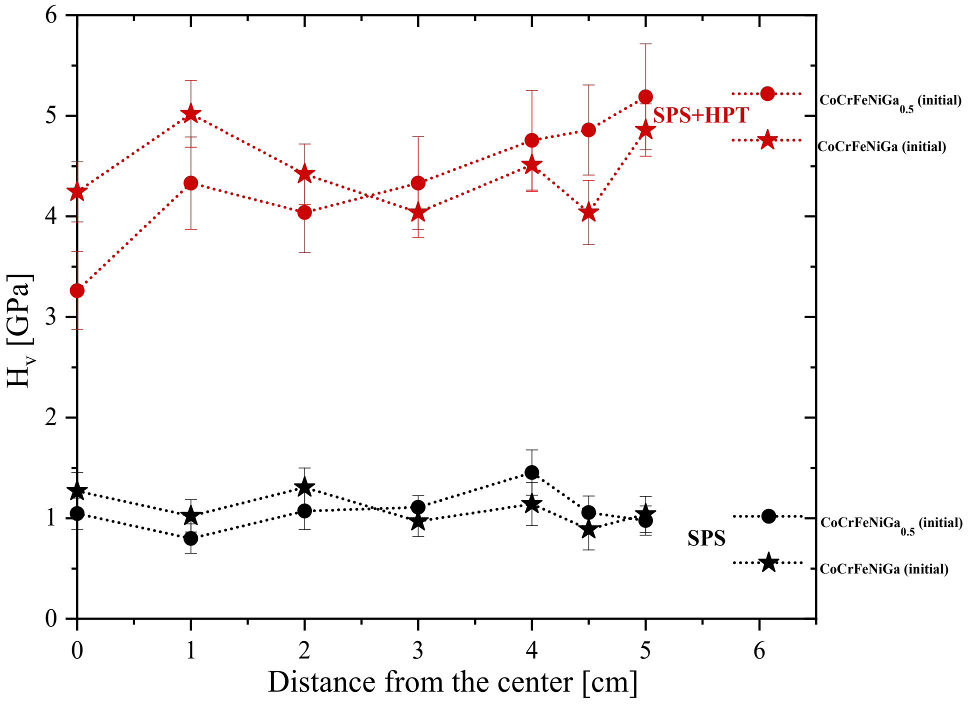

Figure S5. Dependence of the Vickers micro-hardness on the radial distance from the center for SPS-consolidated (black) and subsequently HPT-deformed (red) $CoCrFeNiGa_x$ (x = 0.5, 1.0) samples from elemental powder blends

H_v of the SPS-consolidated $CoCrFeNiGa_x$ (x = 0.5, 1.0) multiphase alloys increased by a factor of 4 after HPT treatment (Figure S5, red). We attribute this to a reduction of the crystallite size and a substantial increase of microstrains (for details, see *section S1*). The variation of H_v from center to the edge is still present.

S3. SEM/EDX results of CoCrFeNiGa bulk HEA after HPT

SEM/EDX results of the HPT-deformed CoCrFeNiGa HEAs show that the microstructural changes of the center (Figure S6) and the edge (Figure S7) of the sample are negligible, and the chemical composition with the uniform distribution of the elements are preserved.

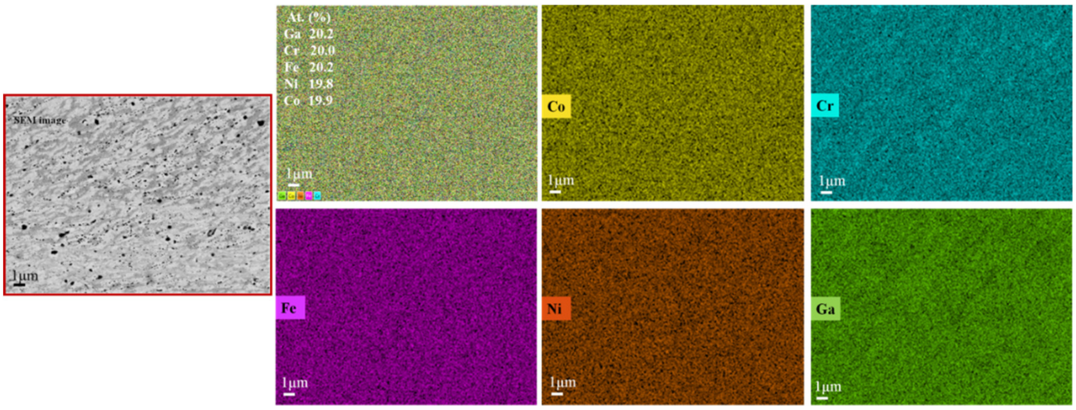

Figure S6 Representative SEM image (cross-section, center of the sample) and compositional EDX mapping results for bulk CoCrFeNiGa sample (HEBM 180+10min/900 rpm, SPS 1073K, 50MPa) after HPT.

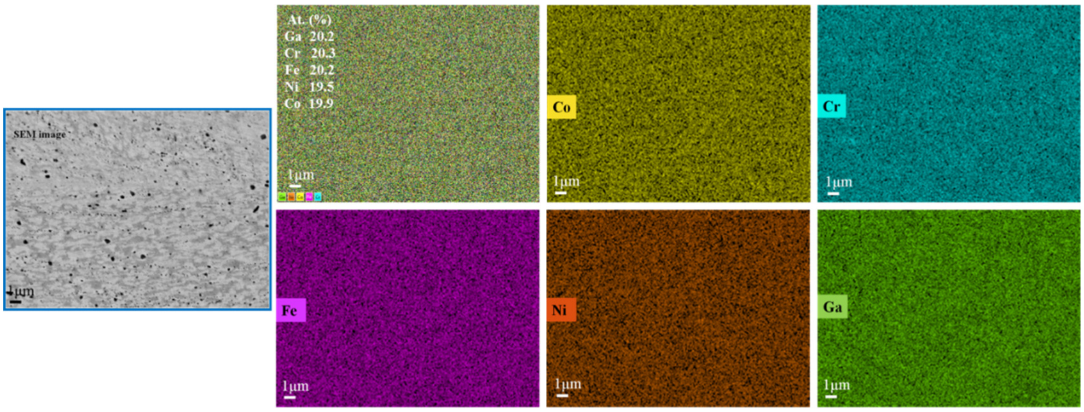

Figure S7 Representative SEM image (cross-section, edge of the sample) and compositional EDX mapping results for bulk CoCrFeNiGa sample (HEBM 180+10min/900 rpm, SPS 1073K, 50MPa) after HPT.
